# Minimally Invasive Oncologic Upper Gastrointestinal Surgery can be Performed Safely on all Weekdays: A Nationwide Cohort Study

**DOI:** 10.1007/s00268-021-06160-x

**Published:** 2021-05-25

**Authors:** Daan M. Voeten, Arthur K. E. Elfrink, Suzanne S. Gisbertz, Jelle P. Ruurda, Richard van Hillegersberg, Mark I. van Berge Henegouwen

**Affiliations:** 1 Department of Surgery, Amsterdam UMC, University of Amsterdam, Cancer Center Amsterdam, Room G6-250, Meibergdreef 9, 1105 AZ, Amsterdam, the Netherlands; 2 Scientific Bureau, Dutch Institute for Clinical Auditing, Leiden, the Netherlands; 3 Department of Surgery, University Medical Center Groningen, Groningen, the Netherlands; 4 Department of Surgery, University Medical Center Utrecht, Utrecht, the Netherlands

## Abstract

**Background:**

Existing literature suggests deteriorating surgical outcome of esophagogastric surgery as the week progresses. However, these studies were conducted in the pre-centralization and pre-minimally invasive era. In addition, they failed to correct for fixed weekdays of esophagogastric cancer surgery among hospitals. This study aimed to describe the impact of weekday of minimally invasive upper gastrointestinal surgery on short-term surgical outcomes.

**Methods:**

All patients registered in the Dutch Upper Gastrointestinal Cancer Audit who underwent curative minimally invasive esophageal or gastric carcinoma surgery in 2015–2019, were included in this nationwide cohort study. Using multilevel multivariable logistic regression, the impact of weekday of surgery on 14 short-term surgical outcomes was investigated. To correct for interhospital variance in fixed weekday(s) of surgery multilevel analyses was used. Results were adjusted for patient, tumor, and treatment characteristics using multivariable logistic regression analyses.

**Results:**

This study included 4,102 patients undergoing minimally invasive upper gastrointestinal surgery (2,968 esophageal cancer and 1,134 gastric cancer patients). Weekday of surgery did not impact postoperative complications, severe postoperative complications, surgical/technical complications, medical complications, anastomotic leakage, complicated postoperative course, failure to rescue, surgical radicality, lymph node yield, 30-day/in-hospital mortality, reinterventions, length of ICU stay, 30-day readmission, and textbook outcome after neither esophageal cancer nor gastric cancer surgery.

**Conclusions:**

Minimally invasive esophagogastric surgery can be performed safely on all weekdays with respect to short-term surgical outcomes, which is important information for operation room scheduling.

**Supplementary Information:**

The online version contains supplementary material available at 10.1007/s00268-021-06160-x.

## Introduction

Gastric and esophageal carcinoma are the third and sixth leading causes of cancer-related mortality worldwide [1]. Surgical resection combined with (neo)adjuvant chemo(radio)therapy is the cornerstone of curative treatment [2–4]. Upper gastrointestinal resections are invasive procedures with overall complication rates around 65% and 42% after esophagectomy and gastrectomy, respectively [5]. These technically complex procedures require specialized knowledge and skill, experience and concentration. A Swedish nationwide study hypothesized that surgical team precision deteriorates as the week progresses since they found worse overall survival after esophagectomy on Wednesday–Friday compared to Monday–Tuesday [6]. The study suggested that this weekday effect was aggravated by centralization as high-volume surgeons perform several exhaustive esophagectomies per week. Another explanation might be reduced staffing or less experienced surgeons on-call during the weekends. However, a Dutch study group found no association between weekday of surgery and surgical outcomes after esophagogastric surgery [7, 8]. Therefore, results on the weekday effect of esophagogastric cancer surgery are inconclusive. It is, however, important information for operation room scheduling.

The studies referred to above were conducted largely in the pre-centralization and pre-minimally invasive era. Minimally invasive surgery might require even more concentration and surgical team precision than its open equivalent. Additionally, none of these studies [6–8] accounted for differences in day of the week on which hospitals routinely perform upper gastrointestinal surgery. This is vital as significant hospital variation in outcomes after upper gastrointestinal surgery exists [9]. In addition, we believe short-term outcomes (like surgical/technical complications) are better proxies for surgeon accuracy and the quality of early postoperative care (e.g., failure to rescue; a proxy for early identification; and treatment of postoperative complications) than long-term survival which is multifactorial.

Therefore, this study aimed to describe the impact of weekday of surgery, corrected for interhospital variance in fixed weekday(s) of surgery, on short-term surgical outcomes after minimally invasive esophagectomy and gastrectomy.

## Materials and methods

### Study design

This population-based cohort study used data from the Dutch Upper Gastrointestinal Cancer Audit (DUCA). Since 2011, this compulsory audit registers all esophagogastric cancer patients undergoing surgery with the intention of resection in the Netherlands [10]. In previous verification, completeness was estimated at 99.2% and outcome measure accuracy at 95.3–100% [11]. This study’s protocol received approval from the DUCA scientific committee. Dutch law did not require ethical review or informed consent.

### Patient selection

All patients undergoing curative, minimally invasive, esophagogastric cancer surgery between Jan 1, 2015, and Dec 31, 2019, were considered for inclusion. This timeframe was chosen to minimize selection bias; from 2015 onward the majority of both gastric and esophageal resections was performed on a minimally invasive basis [12]. In addition, hospital volumes stabilized in recent years as a result of centralization of upper gastrointestinal surgery in the Netherlands [13]. Converted procedures were included on an intention-to-treat basis. Patients undergoing emergency surgery or surgery during the weekend, were excluded. In addition, to review the current Dutch situation, patients were excluded when undergoing surgery in hospitals that stopped performing esophagogastric cancer surgery before 2019.

### Primary outcome measures

The impact of weekday of surgery was investigated for the following surgical outcomes: (1) severe postoperative complications (Clavien–Dindo ≥ IIIa) [14], (2) 30-day/in-hospital mortality (i.e., mortality during the primary admission or, in case of discharge, until 30 days postoperatively), (3) textbook outcome [9] (R0 resection, ≥ 15 lymph nodes, hospital stay < 21 days, and no severe intra- or postoperative complication, readmission (to the ICU), or mortality).

### Secondary outcome measures

The following secondary outcome measures were investigated: (1) postoperative complications (any Clavien–Dindo) [14], (2) surgical/technical complications (includes: chyle leakage, anastomotic leakage, gastric tube necrosis, recurrent nerve injury, iatrogenic intestinal/tracheal/bronchial injury, persistent air leakage requiring drainage > 10 days, extraluminal postoperative bleeding, and intraoperative complications), (3) medical complications (all postoperative complications not considered surgical/technical), (4) anastomotic leakage, (5) complicated postoperative course [10] (complication leading to a reintervention, 30-day/in-hospital mortality, or a hospital stay > 21 days), (6) failure to rescue [15] (a complicated postoperative course leading to 30-day/in-hospital mortality), (7) surgical radicality (R0 resection) [16], (8) lymph node yield (< 15 or ≥ 15)[17], (9) surgical/endoscopic reintervention, (10) length of ICU stay (< 2 days or ≥ 2 days), and (11) 30-day readmission.

### Variables for analyses

This study investigated the impact of each of the five weekdays separately, with Monday as reference. In addition, to investigate the hypothesis of decreased surgical precision as the week progresses, Friday and Monday were analyzed separately (Monday versus Tuesday–Friday, and Friday versus Monday–Thursday). To enable comparison with existing literature even though this categorization is arbitrary, additional analysis was performed after dichotomizing weekdays in conformity with previous literature: Monday–Tuesday versus Wednesday–Friday [6–8].

Several variables were used to adjust for baseline characteristics in investigating the association between weekday of surgery and outcomes. These variables included: sex (male, female), age in years (< 65, 65–75, > 75), preoperative weight loss in kilograms (none, 1–5, 6–10, > 10), BMI (< 20, 20–25, 26–30, > 30), Charlson Comorbidity Index [18] (0, 1, 2 +), ASA score (I-II, III +), previous upper gastrointestinal surgery (no, yes), tumor location (esophageal: intrathoracic, gastro-esophageal junction; gastric: corpus, fundus, antrum, pylorus, total stomach, rest stomach, or anastomosis), clinical tumor stage (T0-2, T3-4, Tx), clinical node stage (N0, N + , Nx), neoadjuvant therapy (esophageal: chemoradiotherapy, chemotherapy, none; gastric: chemotherapy, none, other), hospital volume (≤ 40, > 40), year of surgery (2015 to 2019), and type of resection (esophageal: transthoracic (includes both McKeown and Ivor-Lewis procedures), transhiatal; gastric: total, partial gastrectomy). The following variables were added for esophageal carcinoma only: histology (adenocarcinoma, squamous cell carcinoma, other), anastomotic location (intrathoracic, cervical, other), and salvage surgery (no, yes). In the DUCA, salvage surgery is defined as surgery after previous definitive chemoradiotherapy without initial intent of surgical resection. There are no restrictions as to what definitive scheme is used or the length of the interval between definitive chemoradiotherapy and surgery.

### Ancillary support

In the Netherlands, esophagectomy and gastrectomy patients are generally extubated immediately after surgery. After esophagectomy, protocol length of postoperative ICU stay varies from 0 to 2 days among Dutch hospitals [19]. Standard procedure after gastrectomy is not to admit patients to the ICU. In general, physiotherapists and dieticians are part of the treatment team in the early postoperative phase of all esophagectomy and gastrectomy patients.

### Statistical analyses

All analyses were performed separately for esophageal cancer (including gastro-esophageal junction) and gastric cancer. Baseline characteristics were compared between patients undergoing surgery on each weekday using descriptive statistics. Multilevel multivariable logistic regression analyses investigated the impact of weekday of surgery, categorized in the four ways described above, on the 3 primary and 11 secondary outcomes. The two-level random effect accounted for differences in (fixed) days of the week on which hospitals routinely perform esophagogastric surgery. All baseline characteristics described above were added to the multivariable model. In case the degrees of freedom were insufficient for the entire correction model (i.e., < 10 (non)-events per category in the model), only relevant confounders were added. Factors changing any of the ORs of the weekday variable by 10% or more were considered relevant confounders [20, 21]. The relevance of the random effect was assessed using the likelihood ratio test.

A two-tailed p value < 0.05 was considered statistically significant. Missing items were analyzed in separate groups if exceeding 5%. Multicollinearity was assessed in all multivariable analyses by calculating the variance inflation factor (VIF). A VIF ≥ 2.5 was considered indicative of multicollinearity. All statistical analyses were performed using R-studio version 3.5.1, The R Foundation for Statistical Computing [22].

### Sensitivity analyses

To validate if exclusion of patients undergoing open surgery did not bias results, sensitivity analyses were performed including only patients undergoing open and hybrid surgery. These analyses investigated the impact of the dichotomized weekday (Monday–Tuesday versus Wednesday–Friday) on the above-mentioned outcome measures. The dichotomization was applied because of the low number of open resections and subsequent small group sizes on separate weekdays.

## Results

A total of 2,968 esophageal cancer patients from 16 hospitals and 1,134 gastric cancer patients from 15 hospitals were included (*Online Resource Fig. 1*). Annual esophagectomy hospital volumes ranged from 17 to 100, and annual gastrectomy volumes ranged from 4 to 44. Baseline characteristics of patients undergoing surgery for esophageal or gastric cancer on each weekday are depicted in Tables 1 and 2, respectively. Esophageal surgery was most frequently performed on Monday and Tuesday. Tumor location, histology, cT and cN stage, type of esophagectomy, anastomotic location, and hospital volume differed significantly between the weekdays (Table 1). Gastric cancer surgery was also most frequently performed on Monday and Tuesday. Except for cN-stage there were no baseline differences between weekdays.

**Table 1:** Baseline characteristics of esophageal cancer patients undergoing surgery on each day of the week

	**Esophageal carcinoma**						
	Monday (*n* = 804) (%)	Tuesday (*n* = 935) (%)	Wednesday (*n* = 203) (%)	Thursday (*n* = 621) (%)	Friday (*n* = 405) (%)	Total (n = 2968) (%)	P value^a^
**Sex** Male Female Missing	633 (79) 171 (21) 0 (0)	748 (80) 186 (20) 1 (0)	153 (75) 50 (25) 0 (0)	467 (75) 154 (25) 0 (0)	311 (77) 94 (23) 0 (0)	2312 (78) 655 (22) 1 (0)	0.159
**Age** < 65 years 65–75 years > 75 years	308 (38) 395 (49) 101 (13)	367 (39) 454 (49) 114 (12)	82 (40) 97 (48) 24 (12)	271 (44) 288 (46) 62 (10)	153 (38) 198 (49) 54 (13)	1181 (40) 1432 (48) 355 (12)	0.553
**Preoperative weight loss** None 1–5 kg 6–10 kg > 10 kg Missing	269 (34) 222 (28) 187 (23) 80 (10) 46 (6)	303 (32) 262 (28) 240 (26) 106 (11) 24 (3)	75 (37) 55 (27) 35 (17) 25 (12) 13 (6)	201 (32) 189 (30) 133 (21) 77 (12) 21 (3)	148 (37) 121 (30) 66 (16) 45 (11) 25 (6)	996 (34) 849 (29) 661 (22) 333 (11) 129 (4)	0.074
**BMI** < 20 20–25 26–30 > 30 Missing	44 (6) 391 (49) 286 (36) 81 (10) 2 (0)	64 (7) 414 (44) 350 (37) 100 (11) 7 (1)	11 (5) 93 (46) 65 (32) 32 (16) 2 (1)	42 (7) 287 (46) 213 (34) 79 (13) 0 (0)	23 (6) 208 (51) 130 (32) 42 (10) 2 (1)	184 (6) 1393 (47) 1044 (35) 334 (11) 13 (0)	0.226
**CCI**^b^ 0 1 2 + Missing	339 (42) 236 (29) 226 (28) 3 (0)	411 (44) 239 (26) 285 (31) 0 (0)	98 (48) 45 (22) 60 (30) 0 (0)	283 (46) 147 (24) 183 (30) 8 (1)	191 (47) 101 (25) 113 (28) 0 (0)	1322 (45) 768 (26) 867 (29) 11 (0)	0.286
**ASA score**^c^ 1–2 3 + Missing	563 (70) 239 (30) 2 (0)	710 (76) 225 (24) 0 (0)	149 (74) 54 (27) 0 (0)	470 (76) 151 (24) 0 (0)	298 (74) 107 (26) 0 (0)	2190 (74) 776 (26) 2 (0)	0.067
**Previous esophageal or gastric surgery** No Yes Missing	781 (97) 21 (3) 2 (0)	918 (98) 13 (1) 4 (0)	119 (98) 2 (1) 2 (1)	608 (98) 12 (2) 1 (0)	402 (99) 3 (1) 0 (0)	2908 (98) 51 (2) 9 (0)	0.134
**Tumor location** Intrathoracic Gastro-esophageal junction Unknown/missing	673 (84) 130 (16) 1 (0)	727 (78) 203 (22) 5 (1)	149 (73) 54 (27) 0 (0)	487 (78) 133 (21) 1 (0)	319 (79) 84 (21) 2 (1)	2355 (79) 604 (20) 9 (0)	**0.004**
**Histology** Adenocarcinoma Squamous cell Unknown/other Missing	646 (80) 131 (16) 23 (3) 4 (1)	744 (80) 157 (17) 23 (3) 11 (1)	174 (86) 22 (11) 4 (2) 3 (2)	470 (76) 135 (22) 11 (2) 5 (1)	301 (74) 80 (20) 9 (2) 15 (4)	2335 (79) 525 (18) 70 (2) 38 (1)	**0.018**
**Clinical tumor stage** T0-2 T3-4 Tx	194 (24) 586 (73) 24 (3)	214 (23) 675 (72) 46 (5)	49 (24) 145 (71) 9 (4)	125 (20) 483 (78) 13 (2)	90 (22) 307 (76) 8 (2)	672 (23) 2196 (74) 100 (3)	**0.022**
**Clinical node stage** N0 N + Nx	280 (35) 508 (63) 16 (2)	347 (37) 533 (57) 55 (6)	83 (41) 115 (57) 5 (3)	229 (37) 380 (61) 12 (2)	157 (39) 237 (59) 11 (3)	1096 (37) 1773 (60) 99 (3)	** < 0.001**
**Neoadjuvant therapy** Chemoradiotherapy Chemotherapy None Other/Missing	718 (89) 45 (6) 41 (5) 0 (0)	818 (88) 64 (7) 53 (6) 0 (0)	164 (81) 20 (10) 18 (9) 1 (1)	531 (86) 40 (6) 48 (8) 2 (0)	348 (86) 28 (7) 27 (7) 2 (2)	2579 (87) 197 (7) 187 (6) 5 (0)	0.115
**Salvage surgery** No Yes Missing	787 (98) 11 (1) 6 (1)	897 (96) 11 (1) 27 (3)	196 (97) 5 (3) 2 (1)	586 (94) 19 (3) 16 (3)	348 (86) 8 (2) 49 (12)	2814 (95) 54 (2) 100 (3)	0.058
**Esophagectomy** Transthoracic Transhiatal Other	730 (91) 55 (7) 19 (2)	773 (83) 139 (15) 23 (3)	157 (77) 36 (18) 10 (5)	557 (90) 49 (8) 15 (2)	355 (88) 40 (10) 10 (3)	2572 (87) 319 (11) 77 (3)	** < 0.001**
**Anastomosis** Intrathoracic Cervical None/other/unknown Missing	400 (50) 387 (48) 11 (1) 6 (1)	545 (58) 371 (40) 9 (1) 10 (1)	89 (44) 100 (49) 11 (5) 3 (2)	372 (60) 228 (37) 11 (2) 10 (2)	204 (50) 167 (41) 10 (3) 24 (6)	1610 (54) 1253 (42) 52 (2) 53 (2)	** < 0.001**
**Volume**^d^ < = 40 > 40	250 (31) 554 (69)	324 (35) 611 (65)	113 (56) 90 (44)	116 (19) 505 (81)	95 (24) 310 (77)	898 (30) 2070 (70)	** < 0.001**
**Year** 2015 2016 2017 2018 2019	136 (17) 162 (20) 181 (23) 164 (20) 161 (20)	155 (17) 155 (17) 204 (22) 204 (22) 217 (23)	45 (22) 45 (22) 30 (15) 35 (17) 48 (24)	105 (17) 106 (17) 127 (21) 151 (24) 132 (21)	59 (15) 70 (17) 94 (23) 92 (23) 90 (22)	500 (17) 538 (18) 636 (21) 646 (22) 648 (22)	0.120

^a^Chi-square or Fisher’s exact test depending on group sizes

^b^Charlson Comorbidity Index

^c^American Society of Anesthesiologists score

^d^Total annual esophageal cancer surgery volume

**Table 2:** Baseline characteristics of gastric cancer patients undergoing surgery on each day of the week

	**Gastric carcinoma**						
	Monday (n = 311) (%)	Tuesday (n = 275) (%)	Wednesday (n = 124) (%)	Thursday (n = 231) (%)	Friday (n = 193) (%)	Total (n = 1134) (%)	P value^a^
**Sex** Male Female	193 (62) 118 (38)	154 (56) 121 (44)	76 (61) 48 (39)	125 (54) 106 (46)	115 (60) 78 (40)	663 (58.5) 471 (41.5)	0.325
**Age** < 65 years 65–75 years > 75 years	105 (34) 102 (33) 104 (33)	77 (28) 100 (36) 98 (36)	39 (32) 45 (36) 40 (32)	54 (23) 97 (42) 80 (35)	48 (25) 84 (44) 61 (32)	323 (29) 428 (38) 383 (34)	0.143
**Preoperative weight loss** None 1–5 kg 6–10 kg > 10 kg Missing	106 (34) 80 (26) 63 (20) 37 (12) 25 (8)	87 (32) 81 (30) 60 (22) 32 (12) 15 (6)	34 (27) 27 (22) 33 (27) 20 (16) 10 (8)	67 (29) 79 (34) 53 (23) 19 (8) 13 (6)	60 (31) 50 (26) 39 (20) 27 (14) 17 (9)	354 (31) 317 (28) 248 (22) 135 (12) 80 (7)	0.319
**BMI** < 20 20–25 26–30 > 30 Missing	24 (8) 157 (51) 93 (30) 34 (11) 3 (1)	23 (8) 137 (50) 79 (29) 33 (12) 3 (1)	11 (9) 77 (62) 23 (19) 12 (10) 1 (1)	22 (10) 124 (54) 60 (26) 23 (10) 2 (1)	9 (5) 103 (53) 49 (25) 29 (15) 3 (2)	89 (8) 598 (53) 304 (27) 131 (12) 12 (1)	0.279
**CCI**^b^ 0 1 2 +	147 (47) 75 (24) 89 (29)	106 (39) 78 (28) 91 (33)	52 (42) 32 (26) 40 (32)	93 (40) 60 (26) 78 (34)	85 (44) 43 (22) 65 (34)	483 (43) 288 (25) 363 (32)	0.581
**ASA score**^c^ 1–2 3 + Missing	205 (66) 105 (34) 1 (0)	164 (60) 111 (40) 0 (0)	78 (63) 46 (37) 0 (0)	153 (66) 78 (34) 0 (0)	121 (63) 72 (37) 0 (0)	721 (64) 412 (36) 1 (0)	0.476
**Previous esophageal or gastric surgery** No Yes Missing	295 (95) 14 (5) 2 (1)	270 (98) 4 (2) 1 (0)	120 (97) 4 (3) 0 (0)	222 (96) 9 (4) 0 (0)	181 (94) 11 (6) 1 (1)	1088 (96) 42 (4) 4 (0)	0.113
**Tumor location** Corpus Fundus Antrum Pylorus Total stomach Rest stomach/anastomosis Unknown/missing	104 (33) 28 (9) 139 (45) 26 (8) 9 (3) 5 (2) 0 (0)	93 (34) 25 (9) 121 (44) 18 (7) 10 (4) 3 (1) 5 (2)	39 (32) 10 (8) 55 (44) 10 (8) 8 (7) 1 (1) 1 (1)	73 (32) 27 (12) 95 (41) 28 (12) 5 (2) 3 (1) 0 (0)	56 (29) 19 (10) 91 (47) 17 (9) 7 (4) 2 (1) 1 (1)	365 (32) 109 (10) 501 (44) 99 (9) 39 (3) 14 (1) 7 (1)	0.860
**Clinical Tumor stage** T0-2 T3-4 Tx	104 (33) 150 (48) 57 (18)	72 (26) 142 (52) 61 (22)	42 (34) 63 (51) 19 (15)	76 (33) 126 (55) 29 (13)	62 (32) 104 (54) 27 (14)	356 (31) 585 (52) 193 (17)	0.109
**Clinical node stage** N0 N + Nx	184 (59) 103 (33) 24 (8)	129 (47) 106 (39) 40 (15)	68 (55) 48 (39) 8 (7)	118 (51) 99 (43) 14 (6)	104 (54) 80 (42) 9 (5)	603 (53) 436 (38) 95 (9)	** < 0.001**
**Neoadjuvant therapy** Chemotherapy None Other/missing	187 (60) 114 (37) 10 (3)	165 (60) 108 (39) 2 (1)	64 (52) 54 (44) 6 (5)	136 (59) 87 (38) 8 (4)	117 (61) 69 (36) 7 (4)	669 (59) 433 (38) 33 (3)	0.606
**Gastrectomy** Total Partial Other	106 (34) 193 (62) 12 (4)	120 (44) 147 (54) 8 (3)	51 (41) 70 (57) 3 (2)	82 (36) 146 (63) 3 (1)	64 (33) 126 (65) 3 (2)	423 (37) 682 (60) 29 (3)	0.061
**Volume**^d^ < = 40 > 40	289 (93) 22 (7)	255 (93) 20 (7)	121 (98) 3 (2)	208 (90 23 (10)	180 (93) 13 (7)	1053 (93) 81 (7)	0.137
**Year** 2015 2016 2017 2018 2019	53 (17) 57 (18) 56 (18) 71 (23) 74 (24)	43 (16) 60 (22) 55 (20) 61 (22) 56 (20)	17 (14) 18 (15) 24 (19) 35 (28) 30 (24)	39 (17) 36 (16) 45 (20) 48 (21) 63 (27)	16 (8) 40 (21) 33 (17) 54 (28) 50 (26)	168 (15) 211 (19) 213 (19) 269 (24) 273 (24)	0.268

^a^Chi-square or Fisher’s exact test depending on group sizes

^b^Charlson Comorbidity Index

^c^American Society of Anesthesiologists score

^d^Total annual gastric cancer surgery volume

### Short-term outcomes after esophageal and gastric cancer surgery

The incidence of each of the 14 short-term surgical outcomes after esophageal and gastric cancer surgery is presented in Table 3. Severe complications, short-term mortality and textbook outcome rates after esophagectomy were 31%, 2.6% and 47%, respectively, and 19%, 3.3% and 54% after gastrectomy.

**Table 3:** Short-term surgical outcomes after minimally invasive esophageal and gastric cancer surgery in 2015 to 2019

	Esophageal carcinoma	Gastric carcinoma
	Total N = 2968	Total N = 1134
Postoperative complications (yes)	1928 (65.0%)	463 (40.8%)
Severe complications^a^ (yes)	912 (30.7%)	216 (19.0%)
Technical complications^b^ (yes)	1033 (34.8%)	150 (13.2%)
Medical complications^c^ (yes)	988 (33.3%)	340 (30.0%)
Anastomotic leakage (yes)	588 (19.8%)	97 (8.6%)
Complicated postoperative course^d^ (yes)	912 (30.7%)	230 (20.3%)
Failure to rescue^e^ (yes)	76 (8.3%)	36 (15.7%)
Surgical radicality (micro- and macroscopically radical)	2839 (95.7%)	1035 (91.3%)
Resected lymph nodes (≥ 15)	2636 (88.8%)	985 (86.9%)
Reintervention (yes)	783 (26.4%)	211 (18.6%)
Length of ICU stay (≥ 2 days)	1462 (49.3%)	132 (11.6%)
30-day/in-hospital mortality (yes)	78 (2.6%)	37 (3.3%)
30-day readmission (yes)	451 (15.2%)	147 (13.0%)
Textbook outcome^f^ (yes)	1404 (47.3%)	615 (54.2%)

^a^Clavien–Dindo grade III or higher

^b^Includes: postoperative bleeding (excluding intraluminal), recurrent nerve injury, iatrogenic intestinal injury, gastric tube necrosis, iatrogenic tracheal or bronchial injury, persistent air leakage requiring drainage > 10 days postoperatively, chyle leakage, anastomotic leakage, intraoperative complications

^c^All postoperative complications not mentioned in b

^d^Postoperative complication leading to a reintervention, mortality, or prolonged length of hospital stay (> 21 days)

^e^Patients with a complicated postoperative course^d^ eventually dying in hospital or in first 30 days postoperatively

^f^Patients undergoing a radical, curative resection with at least 15 resected lymph nodes, without intraoperative complication, severe postoperative complication^a^, reintervention, readmission (to the ICU), mortality, and a length of hospital stay shorter than 21 days

### The impact of weekday of surgery on outcomes

When analyzing all weekdays separately, there were no statistically significant differences in severe complications, short-term mortality and textbook outcome after esophageal nor gastric cancer surgery (Table 4). There were also no significant differences between the separate days of the week in terms of complications, technical complications, medical complications, anastomotic leakage, complicated postoperative course, failure to rescue, surgical radicality, lymph node yield, reinterventions, length of ICU stay, and 30-day readmissions (Online Resource Table 1).

**Table 4:** Impact of weekday of surgery, Monday versus Tuesday, Wednesday, Thursday, and Friday, on primary outcomes

	Weekday	Esophageal carcinoma					Gastric carcinoma				
		*Corrected for*	Outcome/N	OR^a^	95% CI^b^	P value	*Corrected for*	Outcome/N	OR^a^	95% CI^b^	P value
Severe complications^c^ (yes)	Monday (ref) Tuesday Wednesday Thursday Friday	*All*^*d*^	244 / 736 242 / 860 58 / 178 168 / 567 95 / 322	1 0.83 0.95 0.85 0.81	0.65 – 1.05 0.65 – 1.39 0.65 – 1.11 0.59 – 1.11	0.123 0.797 0.234 0.182	*No relevant confounders identified*^*e*^	61 / 305 48 / 266 22 / 122 42 / 229 40 / 189	1 0.88 0.88 0.90 1.07	0.58 – 1.34 0.50 – 1.49 0.58 – 1.39 0.68 – 1.68	0.553 0.643 0.631 0.755
30-day/in-hospital mortality (yes)	Monday (ref) Tuesday Wednesday Thursday Friday	*No relevant confounders identified*^e^	24 / 736 20 / 860 4 / 178 10 / 567 13 / 322	1 0.71 0.69 0.53 1.25	0.38 – 1.29 0.20 – 1.82 0.24 – 1.09 0.61 – 2.44	0.258 0.504 0.098 0.529	*None*^*f*^	10 / 305 11 / 266 3 / 122 8 / 229 5 / 189	1 1.27 0.74 1.06 0.80	0.53 – 3.09 0.16 – 2.47 0.40 – 2.74 0.25 – 2.29	0.594 0.653 0.987 0.686
Textbook outcome^g^ (yes)	Monday (ref) Tuesday Wednesday Thursday Friday	*All*^*d*^	326 / 736 433 / 860 66 / 178 293 / 567 166 / 322	1 1.15 0.75 1.18 1.21	0.91 – 1.45 0.52 – 1.08 0.92 – 1.51 0.91 – 1.61	0.256 0.126 0.204 0.198	*All*^*h,i*^	163 / 305 150 / 266 61 / 122 128 / 229 101 / 189	1 1.21 0.90 1.01 0.99	0.84 – 1.73 0.58 – 1.41 0.69 – 1.46 0.67 – 1.45	0.308 0.656 0.978 0.948

^a^Odds ratio

^b^95% Confidence interval

^c^Clavien–Dindo grade III or higher

^d^Corrected for: gender, age, preoperative weight loss, BMI, Charlson Comorbidity Index, ASA score, previous esophageal or gastric surgery, tumor location, histology, clinical tumor stage, clinical node stage, neoadjuvant therapy, salvage surgery, hospital volume, year of surgery, type of esophagectomy, location of anastomosis, and hospital identification number as random effect factor

^e^Given insufficient number of degrees of freedom for correction for all possible confounders, only confounders leading to a 10% change in OR were included for analyses. Hospital ID as random effect was added to the model in case the log-likelihood ratio test showed a better fit compared to the original univariable model

^f^Preoperative weight loss and type of gastrectomy were confounders, but given the small group sizes and small number of degrees of freedom multivariable regression was not possible. Univariable results are presented

^g^Patients undergoing a radical, curative resection with at least 15 resected lymph nodes, without intraoperative complication, severe postoperative complication^C^, reintervention, readmission (to the ICU), mortality and a length of hospital stay shorter than 21 days

^h^Corrected for: gender, age, preoperative weight loss, BMI, Charlson Comorbidity Index, ASA score, previous esophageal or gastric surgery, tumor location, clinical tumor stage, clinical node stage, neoadjuvant therapy, hospital volume, year of surgery, type of gastrectomy, and hospital identification number as random effect factor

^i^Tumor location was removed due to multicollinearity with type of gastrectomy (variance inflation factor > 2.5)

Also when analyzing Monday and Friday separately, no statistically significant differences in both primary and secondary outcomes were identified (Tables 5 and 6, Online Resource Tables 2&3*)*.

### Additional analyses

For esophageal cancer, there were no significant differences between surgery on Monday–Tuesday and Wednesday–Friday in any of the 14 investigated outcome measures (Online Resource Table 4). For gastric cancer, 30-day readmission rates were higher after surgery on Wednesday–Friday compared to surgery early in the week (OR 1.43, 95%CI [1.01–2.04]).

### Sensitivity analyses

In total, 840 and 620 patients underwent open/hybrid esophagectomy and gastrectomy, respectively. In these cohorts of patients, outcomes did not differ after surgery on Monday–Tuesday or on Wednesday–Friday (Online Resource Table 5).

**Table 5:** Impact of weekday of surgery, Tuesday through Friday versus Monday, on primary outcomes

	Weekday	Esophageal carcinoma					Gastric carcinoma				
		*Corrected for*	Outcome/N	OR^a^	95% CI^b^	P value	*Corrected for*	Outcome/N	OR^a^	95% CI^b^	P value
**Severe complications**^c^ (yes)	Tue-Fri (ref) Mon	*All*^*d*^	563 / 1927 244 / 736	1 1.18	0.96 – 1.46	0.110	*No relevant confounders identified*^*e*^	152 / 806 61 / 305	1 1.08	0.77 – 1.49	0.666
**30-day/in-hospital mortality** (yes)	Tue-Fri (ref) Mon	*No relevant confounders identified*^*e*^	47 / 1927 24 / 736	1 1.35	0.80 – 2.20	0.243	*No relevant confounders identified*^*e*^	27 / 808 10 / 308	1 0.98	0.45 – 1.99	0.960
**Textbook outcome**^f^ (yes)	Tue-Fri (ref) Mon	*All*^*d*^	958 / 1927 326 / 736	1 0.91	0.74 – 1.11	0.350	*All*^*g,h*^	440 / 808 163 / 308	1 0.936	0.72 – 1.28	0.777

^a^Odds ratio

^b^95% Confidence interval

^c^Clavien–Dindo grade III or higher

^d^Corrected for: gender, age, preoperative weight loss, BMI, Charlson Comorbidity Index, ASA score, previous esophageal or gastric surgery, tumor location, histology, clinical tumor stage, clinical node stage, neoadjuvant therapy, salvage surgery, hospital volume, year of surgery, type of esophagectomy, location of anastomosis, and hospital identification number as random effect factor

^e^Given insufficient number of degrees of freedom for correction for all possible confounders, only confounders leading to a 10% change in OR were included for analyses. Hospital ID as random effect was added to the model in case the log-likelihood ratio test showed a better fit compared to the original univariable model

^f^Patients undergoing a radical, curative resection with at least 15 resected lymph nodes, without intraoperative complication, severe postoperative complication^C^, reintervention, readmission (to the ICU), mortality, and a length of hospital stay shorter than 21 days

^g^Corrected for: gender, age, preoperative weight loss, BMI, Charlson Comorbidity Index, ASA score, previous esophageal or gastric surgery, tumor location, clinical tumor stage, clinical node stage, neoadjuvant therapy, hospital volume, year of surgery, type of gastrectomy, and hospital identification number as random effect factor

^h^Tumor location was removed due to multicollinearity with type of gastrectomy (variance inflation factor > 2.5)

**Table 6:** Impact of weekday of surgery, Monday through Thursday versus Friday, on primary outcomes

	Weekday	Esophageal carcinoma					Gastric carcinoma				
		*Corrected for*	Outcome / N	OR^a^	95% CI^b^	P value	*Corrected for*	Outcome / N	OR^a^	95% CI^b^	P value
Severe complications^c^ (yes)	Mon-Thu (ref) Fri	*All*^*d*^	712 / 2341 95 / 322	1 0.90	0.69 – 1.19	0.471	*No relevant confounders identified*^*e*^	173 / 922 40 / 189	1 1.16	0.78 – 1.70	0.445
30-day/in-hospital mortality (yes)	Mon-Thu (ref) Fri	*No relevant confounders identified*^*e*^	58 / 2341 13 / 322	1 1.65	0.86 – 2.96	0.108	*No relevant confounders identified*^*e*^	32 / 922 5 / 189	1 0.75	0.26 – 1.80	0.564
Textbook outcome^f^ (yes)	Mon-Thu (ref) Fri	*All*^*d*^	1118 / 2341 166 / 322	1 1.12	0.87 – 1.44	0.369	*All*^*g,h*^	502 / 922 101 / 189	1 0.95	0.68 – 1.33	0.777

^a^Odds Ratio.

^b^95% Confidence interval.

^c^Clavien-Dindo grade III or higher.

^d^Corrected for: gender, age, preoperative weight loss, BMI, Charlson Comorbidity Index, ASA-score, previous esophageal or gastric surgery, tumor location, histology, clinical Tumor stage, clinical Node stage, neoadjuvant therapy, salvage surgery, hospital volume, year of surgery, type of esophagectomy, location of anastomosis and hospital identification number as random effect factor.

^e^Given insufficient number of degrees of freedom for correction for all possible confounders, only confounders leading to a 10% change in OR were included for analyses. Hospital ID as random effect was added to the model in case the log-likelihood ratio test showed a better fit compared to the original univariable model.

^f^Patients undergoing a radical, curative resection with at least 15 resected lymph nodes, without intraoperative complication, severe postoperative complicationC, reintervention, readmission (to the ICU), mortality and a length of hospital stay shorter than 21 days.

^g^Corrected for: gender, age, preoperative weight loss, BMI, Charlson Comorbidity Index, ASA-score, previous esophageal or gastric surgery, tumor location, clinical Tumor stage, clinical Node stage, neoadjuvant therapy, hospital volume, year of surgery, type of gastrectomy, and hospital identification number as random effect factor.

^h^Tumor location was removed due to multicollinearity with type of gastrectomy (variance inflation factor >2.5)

## Discussion

This nationwide cohort study is the first to investigate the impact of weekday of surgery on short-term outcomes of minimally invasive esophagogastric cancer surgery after statistical correction for differing fixed weekdays of surgery among hospitals. Weekday of surgery did not impact postoperative complications, severe postoperative complications, surgical/technical complications, medical complications, complicated postoperative course, failure to rescue, surgical radicality, 30-day/in-hospital mortality, lymph node yield, reinterventions, length of ICU stay, 30-day readmission, and textbook outcome after neither esophageal nor gastric cancer surgery. However, readmission rates were higher after gastric cancer surgery on Wednesday–Friday compared to Monday–Tuesday.

### Esophageal cancer

A 2016 Swedish study, including 1748 esophageal cancer patients between 1987 and 2010, concluded that surgery performed on Wednesday through Friday was associated with augmented mortality [6]. It is hypothesized that surgical team precision deteriorates later in the week; a well-rested surgeon could focus for longer time periods early in the week. Alertness was expected to decrease as the week progresses leading to inferior oncologic resections. A stronger association was found among high-volume surgeons, and they concluded that centralization might enhance the weekday effect. A subsequent study showed that the survival difference was not attributable to short-term mortality [23]. This study did not report on surgical radicality. In response to the Swedish studies, a Dutch study including 3,840 esophageal cancer patients between 2005 and 2013, was published in 2017 [7]. No association between weekday of surgery and long-term survival, surgical radicality, lymph node yield or 30-day mortality was identified. Both the Swedish and Dutch studies did not correct for differences in day of the week on which hospitals routinely perform upper gastrointestinal surgery. In addition, the large inclusion periods of these studies might have biased results as clinical practice changed over time. Neither study reported on surgical procedure (minimally invasive or open). Given these objections, re-investigating the subject with the addition of several short-term outcome measures in the current study is justified. After proper correction for baseline characteristics and interhospital variance in fixed weekday(s) of surgery, the current study showed comparable short-term surgical outcomes among all weekdays. We believe that short-term surgical outcomes (like surgical/technical complications and radicality) are better proxies for surgeon accuracy than long-term overall survival [24, 25]. Overall or disease-specific survival is multifactorial, and since esophageal cancer treatment is multimodal, other treatment factors like (neo)adjuvant therapy, play an important role. The results of the current study indicate that surgical precision of esophageal cancer surgery does not deteriorate as the week progresses. Another finding is that a well-rested surgeon after the weekend does not have better results compared to the rest of the week (Monday versus Tuesday–Friday). Nor did surgery on Friday lead to inferior results compared to surgery on Monday through Thursday. The Swedish hypothesis of an enhanced weekday effect due to centralization could not be confirmed even though the current study only included patients after hospital volumes were stable [13].

### Gastric cancer

Another Swedish study (including a largely overlapping cohort with the study described above)[6] found survival benefit for surgery performed early in the week among 6,124 patients with esophagogastric cancer [26]. Subgroup analyses for gastric cancer patients found similar results. A 2018 German single-center study found contradictory results [27]. It included 460 gastric cancer patients and found no association between weekday of surgery and long-term survival, radicality, lymph node yield, or short-term mortality. A nationwide Dutch study including 3,776 gastric cancer patients between 2006 and 2014 also found no association between long-term survival and weekday of gastrectomy [8]. It did, however, find lower lymph node yield after surgery later in the week. The current study did not find significant differences in short-term outcomes between weekdays of gastrectomy. Nor could it conform the results by the previous Dutch study that lymph node yield is lower after surgery late in the week. The current study did display significantly higher 30-day readmission rates after gastrectomy later in the week. A previous DUCA study showed higher readmission rates after weekend discharge [28]. This might explain the higher readmission rates after surgery later in the week as median length of hospital stay after gastrectomy approximates 8 days [5]. This hypothesis could currently not be verified as noise was added to date of discharge to ensure anonymity of the dataset.

Some argue that complex surgery should be performed early in the week since postoperative complications usually emerge after one to three days and healthcare services are downscaled in the weekend [29–31]. The current study showed that failure to rescue, which is a proxy for early identification, recognition and treatment of complications, did not diminish during the week for neither esophagectomy nor gastrectomy. One might argue that no association between failure to rescue and weekday could be found since a large part of patients undergoing surgery late in the week will be on the ICU during the weekend. On the ICU, health-care provision is ordinarily continued during the weekend. However, in 51% of esophagectomy patients length of ICU admission was only 0 or 1 days. This was 88% for gastrectomy patients. Additionally, length of ICU stay was similar when undergoing surgery early or later in the week.

Many factors might play a confounding role when comparing surgical outcomes between weekdays. Not only do hospitals have fixed weekdays of esophagogastric surgery, surgeons might also have fixed surgery days. Variation in operation room personnel, residents, and ICU staff might also confound results. In addition, call schedules and associated fatigue might play a role. Unfortunately, as the DUCA does not register data at individual physician level, these factors could not be accounted for in the current study. However, this is the only study that corrected for fixed weekdays of surgery at hospital level. Existing literature failed to correct for hospital difference in day of surgery. Additionally, given the large number of inclusions, we feel that these possible confounders might level out at a population level. Therefore, current study’s results refute previous literature on the subject and suggest that esophagogastric cancer surgery can be performed safely on all days of the week. This is important information that is helpful in operation room planning.

This study excludes open surgery which might have introduced selection bias. Since minimally invasive surgery is the gold standard in the Netherlands, open surgery might be reserved for anticipated difficult surgery. However, by only including minimally invasive surgery results are more uniform and weekdays can be compared fairly. In addition, the sensitivity analyses (including only patients undergoing open surgery) confirmed the absence of a weekday effect in upper gastrointestinal surgery. Another limitation is that a significant proportion of patients is excluded from analyses due to non-curative surgery (e.g., open-close). However, augmented numbers of non-curative surgery might also reflect inferior surgical quality.

## Conclusions

This Dutch nationwide study conducted in the era of centralization, shows surgical precision of minimally invasive esophagogastric cancer surgery does not deteriorate as the week progresses. In addition, there are no signs of inferior early postoperative care late in the week or during the weekend. Therefore, in the Netherlands, upper gastrointestinal surgery can be conducted safely on all weekdays.

## Supplementary Information

Below is the link to the electronic supplementary material.

Online Resource Figure 1. Title: Flowchart of the study.
Online Resource Table 1. Impact of weekday of surgery, Monday versus Tuesday, Wednesday, Thursday, and Friday, on secondary short-term surgical outcomes after oncologic esophagogastric surgery in 2015-2019.
Online Resource Table 2. Impact of weekday of surgery, Tuesday through Friday versus Monday, on secondary short-term surgical outcomes after oncologic esophagogastric surgery in 2015-2019.
Online Resource Table 3. Impact of weekday of surgery, Monday through Thursday versus Friday, on secondary short-term surgical outcomes after oncologic esophagogastric surgery in 2015-2019.
Online Resource Table 4. Impact of weekday of surgery, Monday-Tuesday versus Wednesday-Friday, on short-term surgical outcomes after oncologic esophagogastric surgery in 2015-2019.
Online Resource Table 5. Impact of weekday of surgery, Monday-Tuesday versus Wednesday-Friday, on short-term surgical outcomes after open or hybrid oncologic esophagogastric surgery in 2015-2019.
Supplementary file1 (DOCX 82 kb)

## References

[1] Bray F, Ferlay J, Soerjomataram I (2018). Global cancer statistics 2018: GLOBOCAN estimates of incidence and mortality worldwide for 36 cancers in 185 countries. CA Cancer J Clin.

[2] Cunningham D, Allum WH, Stenning SP (2006). Perioperative chemotherapy versus surgery alone for resectable gastroesophageal cancer. N Engl J Med.

[3] Ychou M, Boige V, Pignon JP (2011). Perioperative chemotherapy compared with surgery alone for resectable gastroesophageal adenocarcinoma: an FNCLCC and FFCD multicenter phase III trial. J Clin Oncol.

[4] van Hagen P, Hulshof MC, van Lanschot JJ (2012). Preoperative chemoradiotherapy for esophageal or junctional cancer. N Engl J Med.

[5] van der Werf LR, Busweiler LA, van Sandick JW (2020). Reporting national outcomes after esophagectomy and gastrectomy according to the esophageal complications consensus group (ECCG). Ann Surg.

[6] Lagergren J, Mattsson F, Lagergren P (2016). Weekday of esophageal cancer surgery and its relation to prognosis. Ann Surg.

[7] Visser E, van Rossum PS, Verhoeven RH (2017). Impact of Weekday of Esophagectomy on Short-term and Long-term Oncological Outcomes: A Nationwide Population-based Cohort Study in the Netherlands. Ann Surg.

[8] Visser E, Brenkman HJ, Verhoeven RH (2017). Weekday of gastrectomy for cancer in relation to mortality and oncological outcomes - A Dutch population-based cohort study. Eur J Surg Oncol.

[9] Busweiler LA, Schouwenburg MG, van Berge Henegouwen MI (2017). Textbook outcome as a composite measure in oesophagogastric cancer surgery. Br J Surg.

[10] Busweiler LA, Wijnhoven BP, van Berge Henegouwen MI (2016). Early outcomes from the Dutch upper gastrointestinal cancer audit. Br J Surg.

[11] van der Werf LR, Voeten SC, van Loe CMM (2019). Data verification of nationwide clinical quality registries. BJS open.

[12] Dutch Institute for Clinical Auditing (DICA) - Annual report of the Dutch Upper gastrointestinal Cancer Audit 2018. Available from: https://dica.nl/jaarrapportage-2018/duca [Accessed 1 February 2021]

[13] van der Werf LR, Cords C, Arntz I (2019). Population-based study on risk factors for tumor-positive resection margins in patients with gastric cancer. Ann Surg Oncol.

[14] Clavien PA, Sanabria JR, Strasberg SM (1992). Proposed classification of complications of surgery with examples of utility in cholecystectomy. Surgery.

[15] Busweiler LA, Henneman D, Dikken JL (2017). Failure-to-rescue in patients undergoing surgery for esophageal or gastric cancer. Eur J Surg Oncol.

[16] College of American Pathologists (2016). Protocol for the examination of specimens from patients with carcinoma of the esophagus. College of American Pathologists: Northfield

[17] van der Werf LR, Dikken JL, van Berge Henegouwen MI (2018). A population-based study on lymph node retrieval in patients with esophageal cancer: results from the Dutch upper gastrointestinal cancer audit. Ann Surg Oncol.

[18] Charlson ME, Pompei P, Ales KL (1987). A new method of classifying prognostic comorbidity in longitudinal studies: development and validation. J Chronic Dis.

[19] Voeten DM, van der Werf LR, Gisbertz SS, et al. (2021) Postoperative intensive care unit stay after minimally invasive esophagectomy shows large hospital variation. Results from the Dutch Upper Gastrointestinal Cancer Audit. *Eur J Surg Oncol*. S0748–7983(21)00005–6 10.1016/j.ejso.2021.01.005 33485673

[20] Walter S, Tiemeier H (2009). Variable selection: current practice in epidemiological studies. Eur J Epidemiol.

[21] Greenland S (1989). Modeling and variable selection in epidemiologic analysis. Am J Public Health.

[22] R Core Team (2019). R: A language and environment for statistical computing. R Foundation for Statistical Computing, Vienna, Austria. URL https://www.R-project.org/

[23] Lagergren J, Mattsson F, Lagergren P (2016). Weekday of oesophageal cancer surgery in relation to early postoperative outcomes in a nationwide Swedish cohort study. BMJ Open..

[24] Potter J, Fuller C, Ferris M. Local clinical audit: handbook for physicians. Royal college of physicians, Health Care Quality Improvement Partnership

[25] Beck N, van Bommel AC, Eddes EH (2020). The Dutch institute for clinical auditing: achieving codman's dream on a nationwide basis. Ann Surg.

[26] Lagergren J, Mattsson F, Lagergren P (2017). Weekday of cancer surgery in relation to prognosis. Br J Surg.

[27] Berlth F, Messerle K, Plum PS (2018). Impact of the weekday of surgery on outcome in gastric cancer patients who underwent d2-gastrectomy. World J Surg.

[28] Voeten DM, van der Werf LR, van Sandick JW et al. (2020) Length of hospital stay after uncomplicated esophagectomy hospital variation shows room for nationwide improvement. Surg Endosc. Doi: 10.1007/s00464-020-08103-4 10.1007/s00464-020-08103-4 PMC8523439 33104919

[29] Aylin P, Alexandrescu R, Jen MH, et al. (2013) Day of week of procedure and 30 day mortality for elective surgery: retrospective analysis of hospital episode statistics. *BMJ*. 346:f2424 10.1136/bmj.f2424 PMC3665889 23716356

[30] Zare MM, Itani KM, Schifftner TL (2007). Mortality after nonemergent major surgery performed on Friday versus Monday through Wednesday. Ann Surg.

[31] Thompson JS, Baxter BT, Allison JG (2003). Temporal patterns of postoperative complications. Arch Surg.

