# Supplementary material for: Minimally Invasive Oncologic Upper Gastrointestinal Surgery can be Performed Safely on all Weekdays: A Nationwide Cohort Study
Source: World J Surg. 2021 May 25;45(9):2816–29. doi: 10.1007/s00268-021-06160-x (PMC8321995; doi:10.1007/s00268-021-06160-x)
Supplement: Supplementary file 1 — Online Resource Figure 1. Title: Flowchart of the study. Online Resource Table 1. Impact of weekday of surgery, Monday versus Tuesday, Wednesday, Thursday, and Friday, on secondary short-term surgical outcomes after oncologic esophagogastric surgery in 2015-2019. Online Resource Table 2. Impact of weekday of surgery, Tuesday through Friday versus Monday, on secondary short-term surgical outcomes after oncologic esophagogastric surgery in 2015-2019. Online Resource Table 3. Impact of weekday of surgery, Monday through Thursday versus Friday, on secondary short-term surgical outcomes after oncologic esophagogastric surgery in 2015-2019. Online Resource Table 4. Impact of weekday of surgery, Monday-Tuesday versus Wednesday-Friday, on short-term surgical outcomes after oncologic esophagogastric surgery in 2015-2019. Online Resource Table 5. Impact of weekday of surgery, Monday-Tuesday versus Wednesday-Friday, on short-term surgical outcomes after open or hybrid oncologic esophagogastric surgery in 2015-2019. Supplementary file1 (DOCX 82 kb) [file 268_2021_6160_MOESM1_ESM.docx]

**WORLD JOURNAL OF SURGERY**

**Minimally invasive oncologic upper gastrointestinal surgery can be performed safely on all weekdays: a nationwide cohort study.**

Daan M. Voeten^1,2^ MD, Arthur K.E. Elfrink^2,3^ MD, Suzanne S. Gisbertz^1^ MD PhD, Prof. Jelle P. Ruurda^4^ MD PhD, Prof. Richard van Hillegersberg^4^ MD PhD, Prof. Mark I. van Berge Henegouwen^1^ MD PhD, on behalf of the Dutch Upper Gastrointestinal Cancer Audit (DUCA) Group.

1. Department of Surgery, Amsterdam UMC, University of Amsterdam, Cancer Center Amsterdam, Amsterdam, the Netherlands

2. Scientific Bureau, Dutch Institute for Clinical Auditing, Leiden, the Netherlands

3. Department of Surgery, University Medical Center Groningen, Groningen, the Netherlands

4. Department of Surgery, University Medical Center Utrecht, Utrecht, the Netherlands

**Address for correspondence:**

Mark I. van Berge Henegouwen

Department of Surgery Department of Surgery

Amsterdam UMC, location AMC Amsterdam UMC, location AMC

Room G6-250 Room G6-250

Meibergdreef 9 Meibergdreef 9

1105 AZ Amsterdam 1105 AZ Amsterdam

The Netherlands The Netherlands

Tel: 0031 – 20 732 8003 Tel: 0031 – 20 732 8003

E-mail: [m.i.vanbergehenegouwen@amsterdamumc.nl](mailto:m.i.vanbergehenegouwen@amsterdamumc.nl)

**
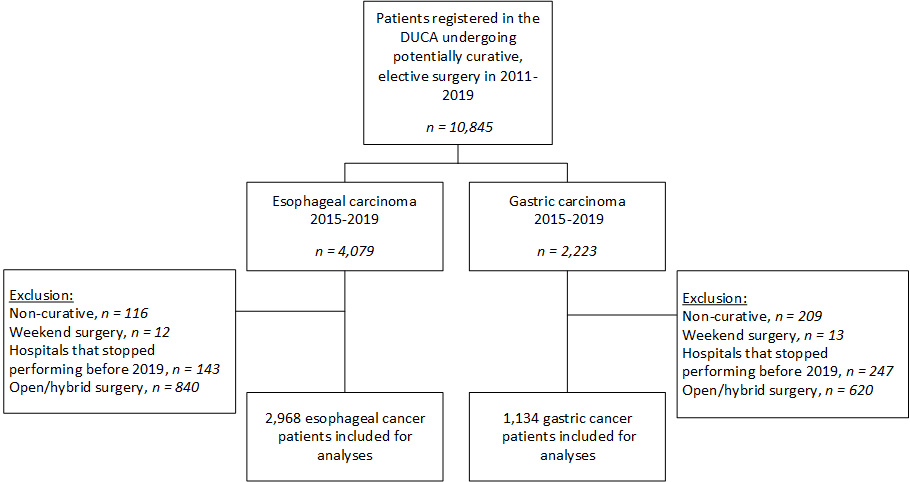
**

| Supplementary file Table 1. Impact of weekday of surgery, Monday versus Tuesday, Wednesday, Thursday, and Friday, on secondary short-term surgical outcomes after oncologic esophagogastric surgery in 2015-2019. | | | | | | | | | | | | |
| --- | --- | --- | --- | --- | --- | --- | --- | --- | --- | --- | --- | --- |
|  |  |  | Esophageal carcinoma | | | |  |  | Gastric carcinoma | | | |
|  |  |  |  | | | |  |  |  | | | |
|  | Weekday | *Corrected for* | Outcome/N | OR^a^ | 95% CI^b^ | P-value |  | *Corrected for* | Outcome/N | OR^a^ | 95% CI^b^ | P-value |
| **Complications** (yes) | Monday (ref)  Tuesday  Wednesday  Thursday  Friday | *All^c^* | 490 / 736  548 / 860  114 / 178  346 / 567  213 / 322 | 1  0.94  0.93  0.80  1.00 | 0.74 – 1.20  0.64 – 1.36  0.62 – 1.05  0.73 – 1.37 | 0.622  0.726  0.110  0.991 |  | *All^d,e^* | 130 / 305  104 / 266  52 / 122  86 / 229  85 / 189 | 1  0.75  0.96  0.80  1.13 | 0.52 – 1.10  0.60 – 1.51  0.54 – 1.18  0.76 – 1.69 | 0.144  0.844  0.264  0.542 |
| **Technical complications^f^**  (yes) | Monday (ref)  Tuesday  Wednesday  Thursday  Friday | *All^c^* | 262 / 736  285 / 860  66 / 178  177 / 567  121 / 322 | 1  0.87  1.13  0.89  1.08 | 0.67 – 1.12  0.78 – 1.65  0.67 – 1.16  0.79 – 1.47 | 0.280  0.519  0.380  0.631 |  | *No relevant confounders identified*^g^ | 39 / 305  39 / 266  16 / 122  24 / 229  28 / 189 | 1  1.17  1.03  0.80  1.19 | 0.73 – 1.89  0.54 – 1.89  0.46 – 1.36  0.70 – 2.00 | 0.516  0.927  0.414  0.523 |
| **Medical complications**^h^  (yes) | Monday (ref)  Tuesday  Wednesday  Thursday  Friday | *All^c^* | 256 / 736  284 / 860  56 / 178  182 / 567  104 / 322 | 1  1.01  0.85  0.84  0.92 | 0.78 – 1.30  0.58 – 1.26  0.64 – 1.10  0.67 – 1.26 | 0.950  0.427  0.198  0.595 |  | *No relevant confounders identified*^g^ | 99 / 305  74 / 266  41 / 122  64 / 229  60 / 189 | 1  0.80  1.05  0.81  0.97 | 0.56 – 1.15  0.67 – 1.64  0.55 – 1.17  0.65 – 1.43 | 0.229  0.820  0.263  0.869 |
| **Anastomotic leakage**  (yes) | Monday (ref)  Tuesday  Wednesday  Thursday  Friday | *All^c^* | 143 / 736  181 / 860  35 / 178  90 / 567  67 / 322 | 1  1.04  1.27  0.96  1.24 | 0.77 – 1.40  0.81 – 2.00  0.69 – 1.34  0.86 – 1.79 | 0.801  0.291  0.814  0.258 |  | *Type of gastrectomy*^g^ | 22 / 305  24 / 266  8 / 122  20 / 229  19 / 189 | 1  1.16  0.84  1.27  1.54 | 0.63 – 2.16  0.34 – 1.91  0.66 – 2.43  0.79 – 2.97 | 0.640  0.695  0.463  0.200 |
| **Complicated postoperative course**^i^  (yes) | Monday (ref)  Tuesday  Wednesday  Thursday  Friday | *All^c^* | 247 / 736  242 / 860  59 / 178  163 / 567  96 / 322 | 1  0.87  0.99  0.85  0.85 | 0.68 – 1.10  0.69 – 1.44  0.65 – 1.10  0.62 – 1.15 | 0.250  0.977  0.222  0.282 |  | *No relevant confounders identified*^g^ | 64 / 305  48 / 266  24 / 122  49 / 229  41 / 189 | 1  0.83  0.92  1.03  1.04 | 0.54 – 1.26  0.54 – 1.54  0.67 – 1.56  0.67 – 1.62 | 0.378  0.762  0.908  0.851 |
| **Failure to rescue^j^**  (yes) | Monday (ref)  Tuesday  Wednesday  Thursday  Friday | *Location of anastomosis*^g^ | 23 / 247  20 / 242  4 / 59  9 / 163  13 / 96 | 1  0.92  0.75  0.60  1.55 | 0.90 – 1.72  0.06 – 2.07  0.48 – 1.29  0.73 – 3.17 | 0.789  0.611  0.205  0.238 |  | *None^k^* | 10 / 64  11 / 48  3 / 24  7 / 49  5 / 41 | 1  1.58  0.76  0.88  0.74 | 0.60 – 4.15  0.16 – 2.77  0.30 – 2.50  0.21 – 2.26 | 0.350  0.694  0.816  0.603 |
| **Surgical radicality**  (micro- and macroscopically radical) | Monday (ref)  Tuesday  Wednesday  Thursday  Friday | *No relevant confounders identified*^G^ | 701 / 736  824 / 860  167 / 178  546 / 567  305 / 322 | 1  1.45  0.69  1.47  0.93 | 0.86 – 2.47  0.35 – 1.47  0.82 – 2.73  0.50 – 1.79 | 0.170  0.308  0.210  0.816 |  | *No relevant confounders identified*^g^ | 281 / 305  237 / 266  108 / 122  216 / 229  174 / 189 | 1  0.77  0.65  1.54  1.05 | 0.42 – 1.42  0.32 – 1.37  0.74 – 3.36  0.52 – 2.19 | 0.404  0.242  0.258  0.897 |
| **Resected lymph nodes**  (≥15) | Monday (ref)  Tuesday  Wednesday  Thursday  Friday | *Hospital volume, esopha-gectomy type, random effect of hospital identification number*^g^ | 645 / 736  767 / 860  143 / 178  626 / 567  293 / 322 | 1  1.03  0.84  0.94  0.89 | 0.69 – 1.54  0.50 – 1.41  0.58 – 1.51  0.53 – 1.49 | 0.896  0.500  0.786  0.647 |  | *Random effect of hospital identification number^g^* | 263 / 305  238 / 266  99 / 122  202 / 229  163 / 189 | 1  1.18  0.61  1.03  1.04 | 0.67 – 2.09  0.33 – 1.11  0.58 – 1.81  0.59 – 1.82 | 0.568  0.107  0.921  0.900 |
| **Reintervention**  (yes) | Monday (ref)  Tuesday  Wednesday  Thursday  Friday | *All^c^* | 215 / 736  205 / 860  47 / 178  140 / 567  84 / 322 | 1  0.81  0.87  0.80  0.83 | 0.63 – 1.05  0.59 – 1.28  0.61 – 1.06  0.61 – 1.14 | 0.108  0.475  0.117  0.257 |  | *No relevant confounders identified*^g^ | 63 / 305  41 / 266  21 / 122  47 / 229  36 / 189 | 1  0.70  0.80  0.99  0.91 | 0.45 – 1.08  0.45 – 1.36  0.65 – 1.51  0.57 – 1.42 | 0.106  0.419  0.970  0.664 |
| **Length of ICU stay**  (≥2 days) | Monday (ref)  Tuesday  Wednesday  Thursday  Friday | *All^c^* | 443 / 736  306 / 860  108 / 178  285 / 567  162 / 322 | 1  0.85  0.72  0.85  1.03 | 0.64 – 1.13  0.46 – 1.11  0.62 – 1.16  0.72 – 1.47 | 0.262  0.139  0.305  0.871 |  | *No relevant confounders identified*^g^ | 37 / 305  27 / 266  20 / 122  21 / 229  25 / 189 | 1  0.82  1.42  0.73  1.10 | 0.48 – 1.38  0.78 – 2.54  0.41 – 1.28  0.64 – 1.89 | 0.455  0.244  0.278  0.721 |
| **30-day readmission**  (yes) | Monday (ref)  Tuesday  Wednesday  Thursday  Friday | *All^c^* | 117 / 736  115 / 860  36 / 178  86 / 567  3 / 322 | 1  0.87  1.43  1.02  0.81 | 0.64 – 1.18  0.92 – 2.22  0.73 – 1.43  0.54 – 1.23 | 0.376  0.108  0.892  0.331 |  | *No relevant confounders identified*^g^ | 35 / 305  28 / 266  21 / 122  31 / 229  29 / 189 | 1  0.88  1.57  1.21  1.39 | 0.52 – 1.49  0.86 – 2.80  0.72 – 2.03  0.81 – 2.35 | 0.638  0.135  0.470  0.228 |
| a *Odds Ratio*  b *95% Confidence interval*  c *Corrected for: gender, age, preoperative weight loss, BMI, Charlson Comorbidity Index, ASA-score, previous esophageal or gastric surgery, tumors location, histology, clinical Tumor stage, clinical Node stage, neoadjuvant therapy, salvage surgery, hospital volume, year of surgery, type of esophagectomy, location of anastomosis and hospital identification number as random effect factor.*  d *Corrected for: gender, age, preoperative weight loss, BMI, Charlson Comorbidity Index, ASA-score, previous esophageal or gastric surgery, tumor location, clinical Tumor stage, clinical Node stage, neoadjuvant therapy, hospital volume, year of surgery, type of gastrectomy, and hospital identification number as random effect factor.*  e *Tumor location was removed due to multicollinearity with type of gastrectomy (variance inflation factor >2.5)*  f *Includes*: *postoperative bleeding (excluding intraluminal), recurrent nerve injury, iatrogenic intestinal injury, gastric tube necrosis, iatrogenic tracheal or bronchial injury, persistent air leakage requiring drainage > 10 days postoperatively, chyle leakage, anastomotic leakage, intraoperative complications.*  g *Given insufficient number of degrees of freedom for correction for all possible confounders, only confounders leading to a 10% change in OR were included for analyses. Hospital ID as random effect was added to the model in case the log-likelihood ratio test showed a better fit compared to the original univariable model.*  h *All postoperative complications not mentioned in* F*.*  i *Postoperative complication leading to a reintervention, mortality, or prolonged length of hospital stay (>21 days)*  j *Patients with a complicated postoperative course eventually dying in hospital or in first 30-days postoperatively*  k *Age, type of gastrectomy and year of surgery were confounders but given the small group sizes and small number of degrees of freedom multivariable regression was not possible. Univariable results are presented.* | | | | | | | | | | | | |

| Supplementary file Table 2. Impact of weekday of surgery, Tuesday through Friday versus Monday, on secondary short-term surgical outcomes after oncologic esophagogastric surgery in 2015-2019. | | | | | | | | | | | | |
| --- | --- | --- | --- | --- | --- | --- | --- | --- | --- | --- | --- | --- |
|  |  |  | Esophageal carcinoma | | | |  |  | Gastric carcinoma | | | |
|  |  |  |  | | | |  |  |  | | | |
|  | Weekday | *Corrected for* | Outcome/N | OR^a^ | 95% CI^b^ | P-value |  | *Corrected for* | Outcome/N | OR^a^ | 95% CI^b^ | P-value |
| **Complications** (yes) | Tue-Fri (ref)  Mon | *All^c^* | 1221 / 1927  490 / 736 | 1  1.10 | 0.89 – 1.36 | 0.379 |  | *All^d,e^* | 327 / 806  130 / 305 | 1  1.13 | 0.84 – 1.52 | 0.414 |
| **Technical complications**^f^  (yes) | Tue-Fri (ref)  Mon | *All^c^* | 649 / 1927  262 / 736 | 1  1.06 | 0.86 – 1.31 | 0.587 |  | *No relevant confounders identified^g^* | 107 / 806  39 / 305 | 0.96 | 0.64 – 1.41 | 0.830 |
| **Medical complications^h^**  (yes) | Tue-Fri (ref)  Mon | *All^c^* | 626 / 1927  256 / 736 | 1  1.09 | 0.88 – 1.35 | 0.447 |  | *All^d,e^* | 239 / 806  99 / 305 | 1  1.17 | 0.86 – 1.58 | 0.321 |
| **Anastomotic leakage**  (yes) | Tue-Fri (ref)  Mon | *All^c^* | 373 / 1927  143 / 736 | 1  0.92 | 0.72 – 1.19 | 0.545 |  | *No relevant confounders identified^g^* | 71 / 806  22 / 305 | 1  0.80 | 0.48 – 1.30 | 0.389 |
| **Complicated postoperative course**^i^  (yes) | Tue-Fri (ref)  Mon | *All^c^* | 560 / 1927  247 / 736 | 1  1.15 | 0.93 – 1.41 | 0.189 |  | *No relevant confounders identified^g^* | 162 / 806  64 / 305 | 1  1.06 | 0.76 – 1.45 | 0.744 |
| **Failure to rescue^j^**  (yes) | Tue-Fri (ref)  Mon | *No relevant confounders identified^g^* | 46 / 560  23 / 247 | 1  1.14 | 0.66 – 1.91 | 0.618 |  | *No relevant confounders identified^g^* | 26 / 162  10 /64 | 1  0.99 | 0.43 – 2.13 | 0.974 |
| **Surgical radicality**  (micro- and macroscopically radical) | Tue-Fri (ref)  Mon | *No relevant confounders identified^g^* | 1842 / 1927  701 / 736 | 1  0.82 | 0.54 – 1.27 | 0.365 |  | *No relevant confounders identified^g^* | 735 / 806  281 / 305 | 1  1.06 | 0.65 – 1.79 | 0.821 |
| **Resected lymph nodes**  (≥15) | Tue-Fri (ref)  Mon | *Random effect of hospital identification number^g^* | 1729 / 1927  645 / 736 | 1  1.06 | 0.76 – 1.48 | 0.737 |  | *Random effect of hospital identification number^g^* | 702 / 806  263 / 305 | 1  1.04 | 0.68 – 1.59 | 0.869 |
| **Reintervention**  (yes) | Tue-Fri (ref)  Mon | *All^c^* | 476 / 1927  215 / 736 | 1  1.22 | 0.99 – 1.51 | 0.068 |  | *No relevant confounders identified^g^* | 145 / 806  63 / 305 | 1  1.19 | 0.85 – 1.65 | 0.310 |
| **Length of ICU stay**  (≥2 days) | Tue-Fri (ref)  Mon | *All^c^* | 861 / 1927  443 / 736 | 1  1.16 | 0.91 – 1.48 | 0.226 |  | *No relevant confounders identified^g^* | 93 / 806  37 / 305 | 1  1.06 | 0.70 – 1.58 | 0.784 |
| **30-day readmission**  (yes) | Tue-Fri (ref)  Mon | *All^c^* | 276 / 1927  117 / 736 | 1  1.03 | 0.80 – 1.34 | 0.803 |  | *No relevant confounders identified^g^* | 109 / 806  35 / 305 | 1  0.84 | 0.55 – 1.25 | 0.406 |
| a *Odds Ratio*  b *95% Confidence interval*  c *Corrected for: gender, age, preoperative weight loss, BMI, Charlson Comorbidity Index, ASA-score, previous esophageal or gastric surgery, tumor location, histology, clinical Tumor stage, clinical Node stage, neoadjuvant therapy, salvage surgery, hospital volume, year of surgery, type of esophagectomy, location of anastomosis and hospital identification number as random effect factor.*  d *Corrected for: gender, age, preoperative weight loss, BMI, Charlson Comorbidity Index, ASA-score, previous esophageal or gastric surgery, tumor location, clinical Tumor stage, clinical Node stage, neoadjuvant therapy, hospital volume, year of surgery, type of gastrectomy, and hospital identification number as random effect factor.*  e *Tumor location was removed due to multicollinearity with type of gastrectomy (variance inflation factor >2.5)*  f *Includes*: *postoperative bleeding (excluding intraluminal), recurrent nerve injury, iatrogenic intestinal injury, gastric tube necrosis, iatrogenic tracheal or bronchial injury, persistent air leakage requiring drainage > 10 days postoperatively, chyle leakage, anastomotic leakage, intraoperative complications.*  g *Given insufficient number of degrees of freedom for correction for all possible confounders, only confounders leading to a 10% change in OR were included for analyses. Hospital ID as random effect was added to the model in case the log-likelihood ratio test showed a better fit compared to the original univariable model.*  h *All postoperative complications not mentioned in F.*  i *Postoperative complication leading to a reintervention, mortality, or prolonged length of hospital stay (>21 days)*  j *Patients with a complicated postoperative course eventually dying in hospital or in first 30-days postoperatively* | | | | | | | | | | | | |

| Supplementary file Table 3. Impact of weekday of surgery, Monday through Thursday versus Friday, on secondary short-term surgical outcomes after oncologic esophagogastric surgery in 2015-2019. | | | | | | | | | | | | |
| --- | --- | --- | --- | --- | --- | --- | --- | --- | --- | --- | --- | --- |
|  |  |  | Esophageal carcinoma | | | |  |  | Gastric carcinoma | | | |
|  |  |  |  | | | |  |  |  | | | |
|  | Weekday | *Corrected for* | Outcome / N | OR^a^ | 95% CI^b^ | P-value |  | *Corrected for* | Outcome / N | OR^a^ | 95% CI^b^ | P-value |
| **Complications** (yes) | Mon-Thu (ref)  Fri | *All*^c^ | 1498 / 2341  213 / 322 | 1  1.10 | 0.84 – 1.44 | 0.490 |  | *All*^d,e^ | 372 / 922  85 / 189 | 1  1.30 | 0.92 – 1.83 | 0.137 |
| **Technical complications**^f^  (yes) | Mon-Thu (ref)  Fri | *All*^c^ | 790 / 2341  121 / 322 | 1  1.16 | 0.89 – 1.52 | 0.258 |  | *No relevant confounders identified^g^* | 118 / 922  28 / 189 | 1  1.18 | 0.75 – 1.83 | 0.455 |
| **Medical complications**^h^  (yes) | Mon-Thu (ref)  Fri | *All*^c^ | 778 / 2341  104 / 322 | 1  0.98 | 0.75 – 1.29 | 0.888 |  | *All*^d,e^ | 278 / 922  60 / 189 | 1  1.14 | 0.80 – 1.63 | 0.470 |
| **Anastomotic leakage**  (yes) | Mon-Thu (ref)  Fri | *All*^c^ | 449 / 2341  67 / 322 | 1  1.22 | 0.89 – 1.67 | 0.225 |  | *Gastectomy type^g^* | 74 / 922  19 / 189 | 1  1.41 | 0.80 – 2.39 | 0.216 |
| **Complicated postoperative course**^i^  (yes) | Mon-Thu (ref)  Fri | *All*^c^ | 711 / 2341  96 / 322 | 1  0.93 | 0.71 – 1.22 | 0.591 |  | *No relevant confounders identified^g^* | 185 / 922  41 / 189 | 1  1.10 | 0.75 – 1.60 | 0.613 |
| **Failure to rescue^j^**  (yes) | Mon-Thu (ref)  Fri | *No relevant confounders identified^g^* | 56 / 711  13 / 96 | 1  1.83 | 0.92 – 3.39 | 0.067 |  | *Gastectomy type^g^* | 31 / 185  5 / 41 | 1  0.76 | 0.24 – 1.97 | 0.593 |
| **Surgical radicality**  (micro- and macroscopically radical) | Mon-Thu (ref)  Fri | *No relevant confounders identified^g^* | 2238 / 2341  305 / 322 | 1  0.78 | 0.46 – 1.42 | 0.389 |  | *No relevant confounders identified^g^* | 842 / 922  174 / 189 | 1  1.11 | 0.62 – 2.15 | 0.733 |
| **Resected lymph nodes**  (≥15) | Mon-Thu (ref)  Fri | *Hospital volume, random effect of hospital identification number^g^* | 2081 / 2341  293 / 322 | 1  0.95 | 0.61 – 1.47 | 0.808 |  | *Random effect of hospital identification number^g^* | 802 / 922  163 / 189 | 1  1.09 | 0.67 – 1.78 | 0.733 |
| **Reintervention**  (yes) | Mon-Thu (ref)  Fri | *All*^c^ | 607 / 2341  84 / 322 | 1  0.96 | 0.72 – 1.27 | 0.757 |  | *No relevant confounders identified^g^* | 172 / 922  36 / 189 | 1  1.03 | 0.68 – 1.51 | 0.900 |
| **Length of ICU stay**  (≥2 days) | Mon-Thu (ref)  Fri | *All*^c^ | 1142 / 2341  162 / 322 | 1  1.17 | 0.87 – 1.58 | 0.299 |  | *Gastrectomy type^g^* | 105 / 922  25 / 189 | 1  1.31 | 0.80 – 2.09 | 0.268 |
| **30-day readmission**  (yes) | Mon-Thu (ref)  Fri | *All*^c^ | 354 / 2341  39 / 322 | 1  0.82 | 0.57 – 1.19 | 0.298 |  | *No relevant confounders identified^g^* | 115 / 922  29 / 189 | 1  1.28 | 0.81 – 1.96 | 0.281 |
| a *Odds Ratio*  b *95% Confidence interval*  c *Corrected for: gender, age, preoperative weight loss, BMI, Charlson Comorbidity Index, ASA-score, previous esophageal or gastric surgery, tumor location, histology, clinical Tumor stage, clinical Node stage, neoadjuvant therapy, salvage surgery, hospital volume, year of surgery, type of esophagectomy, location of anastomosis and hospital identification number as random effect factor.*  d *Corrected for: gender, age, preoperative weight loss, BMI, Charlson Comorbidity Index, ASA-score, previous esophageal or gastric surgery, tumor location, clinical Tumor stage, clinical Node stage, neoadjuvant therapy, hospital volume, year of surgery, type of gastrectomy, and hospital identification number as random effect factor.*  e *Tumor location was removed due to multicollinearity with type of gastrectomy (variance inflation factor >2.5)*  f *Includes*: *postoperative bleeding (excluding intraluminal), recurrent nerve injury, iatrogenic intestinal injury, gastric tube necrosis, iatrogenic tracheal or bronchial injury, persistent air leakage requiring drainage > 10 days postoperatively, chyle leakage, anastomotic leakage, intraoperative complications.*  g *Given insufficient number of degrees of freedom for correction for all possible confounders, only confounders leading to a 10% change in OR were included for analyses. Hospital ID as random effect was added to the model in case the log-likelihood ratio test showed a better fit compared to the original univariable model.*  h *All postoperative complications not mentioned in F.*  i *Postoperative complication leading to a reintervention, mortality, or prolonged length of hospital stay (>21 days)*  j *Patients with a complicated postoperative course eventually dying in hospital or in first 30-days postoperatively* | | | | | | | | | | | | |

| Supplementary file Table 4. Impact of weekday of surgery, Monday-Tuesday versus Wednesday-Friday, on short-term surgical outcomes after oncologic esophagogastric surgery in 2015-2019. | | | | | | | | | | | | |
| --- | --- | --- | --- | --- | --- | --- | --- | --- | --- | --- | --- | --- |
|  |  |  | Esophageal carcinoma | | | |  |  | Gastric carcinoma | | | |
|  |  |  |  | | | |  |  |  | | | |
|  | Weekday | *Corrected for* | Outcome/N | OR^a^ | 95% CI^b^ | P-value |  | *Corrected for* | Outcome/N | OR^a^ | 95% CI^b^ | P-value |
| **Complications** (yes) | Mon-Tue (ref)  Wed-Fri | *All^c^* | 1038 / 1596  673 / 1067 | 1  0.91 | 0.76 – 1.09 | 0.321 |  | *All^d,e^* | 234 / 571  223 / 540 | 1  1.07 | 0.83 – 1.39 | 0.600 |
| **Severe complications**^f^ (yes) | Mon-Tue (ref)  Wed-Fri | *All^c^* | 486 / 1596  321 / 1067 | 1  0.95 | 0.79 – 1.14 | 0.575 |  | *No relevant confounders identified^g^* | 109 / 571  104 / 540 | 1  1.0 | 0.75 – 1.36 | 0.943 |
| **Technical complications**^h^ (yes) | Mon-Tue (ref)  Wed-Fri | *All^c^* | 547 / 1596  364 / 1067 | 1  1.06 | 0.88 – 1.28 | 0.528 |  | *No relevant confounders identified^g^* | 78 / 571  68 / 540 | 1  0.91 | 0.64 – 1.29 | 0.599 |
| **Medical complications**^i^  (yes) | Mon-Tue (ref)  Wed-Fri | *All^c^* | 540 / 1596  342 / 1067 | 1  0.86 | 0.71 – 1.04 | 0.116 |  | *All^d,e^* | 173 / 571  165 / 540 | 1  1.06 | 0.80 – 1.38 | 0.696 |
| **Anastomotic leakage**  (yes) | Mon-Tue (ref)  Wed-Fri | *All^c^* | 324 / 1596  192 / 1067 | 1  1.08 | 0.85 – 1.36 | 0.522 |  | *No relevant confounders identified^g^* | 46 / 571  47 / 540 | 1  1.09 | 0.71 – 1.67 | 0.690 |
| **Complicated postoperative course^j^**  (yes) | Mon-Tue (ref)  Wed-Fri | *All^c^* | 489 / 1596  318 / 1067 | 1  0.94 | 0.78 – 1.14 | 0.539 |  | *No relevant confounders identified^g^* | 112 / 571  114 / 540 | 1  1.10 | 0.82 – 1.47 | 0.536 |
| **Failure to rescue^k^**  (yes) | Mon-Tue (ref)  Wed-Fri | *No relevant confounders identified^g^* | 43 / 489  26 / 318 | 1  0.93 | 0.55 – 1.54 | 0.780 |  | *No relevant confounders identified^g^* | 21 / 112  15 / 114 | 1  0.65 | 0.31 – 1.33 | 0.241 |
| **Surgical radicality**  (micro- and macroscopically radical) | Mon-Tue (ref)  Wed-Fri | *No relevant confounders identified^g^* | 1525 / 1596  1018 / 1067 | 1  0.90 | 0.60 – 1.35 | 0.609 |  | *No relevant confounders identified^g^* | 518 / 571  498 / 540 | 1  1.20 | 0.76 – 1.88 | 0.437 |
| **Resected lymph nodes**  (≥15) | Mon-Tue (ref)  Wed-Fri | *Hospital volume, random effect of hospital identification number^g^* | 1412 / 1596  962 / 1067 | 1  0.92 | 0.68 – 1.24 | 0.578 |  | *Random effect of hospital identification number^g^* | 501 / 571  464 / 540 | 1  0.84 | 0.58 – 1.23 | 0.369 |
| **Reintervention**  (yes) | Mon-Tue (ref)  Wed-Fri | *All^c^* | 420 / 1596  271 / 1067 | 1  0.92 | 0.76 – 1.11 | 0.379 |  | *No relevant confounders identified^g^* | 104 / 571  104 / 540 | 1  1.07 | 0.79 – 1.45 | 0.655 |
| **Length of ICU stay**  (≥2 days) | Mon-Tue (ref)  Wed-Fri | *All^c^* | 749 / 1596  555 / 1067 | 1  0.95 | 0.77 – 1.18 | 0.659 |  | *No relevant confounders identified^g^* | 64 / 571  66 / 540 | 1  1.10 | 0.76 – 1.59 | 0.599 |
| **30-day/in-hospital mortality**  (yes) | Mon-Tue (ref)  Wed-Fri | *No relevant confounders identified^g^* | 44 / 1596  27 / 1067 | 1  0.92 | 0.56 – 1.48 | 0.731 |  | *No relevant confounders identified^g^* | 21 / 571  16 / 540 | 1  0.80 | 0.41 – 1.54 | 0.504 |
| **30-day readmission**  (yes) | Mon-Tue (ref)  Wed-Fri | *All^c^* | 232 / 1596  161 / 1067 | 1  1.12 | 0.88 – 1.42 | 0.351 |  | *No relevant confounders identified^g^* | 63 / 571  81 / 540 | 1  1.43 | 1.01 – 2.04 | **0.046** |
| **Textbook outcome^l^**  (yes) | Mon-Tue (ref)  Wed-Fri | *All^c^* | 759 / 1596  525 / 1067 | 1  1.01 | 0.85 – 1.21 | 0.879 |  | *All^d,e^* | 313 / 571  290 / 540 | 1  0.89 | 0.69 – 1.15 | 0.387 |
| a *Odds Ratio*  b *95% Confidence interval*  c *Corrected for: gender, age, preoperative weight loss, BMI, Charlson Comorbidity Index, ASA-score, previous esophageal or gastric surgery, tumor location, histology, clinical Tumor stage, clinical Node stage, neoadjuvant therapy, salvage surgery, hospital volume, year of surgery, type of esophagectomy, location of anastomosis and hospital identification number as random effect factor.*  d *Corrected for: gender, age, preoperative weight loss, BMI, Charlson Comorbidity Index, ASA-score, previous esophageal or gastric surgery, tumor location, clinical Tumor stage, clinical Node stage, neoadjuvant therapy, hospital volume, year of surgery, type of gastrectomy, and hospital identification number as random effect factor.*  e *Tumor location was removed due to multicollinearity with type of gastrectomy (variance inflation factor >2.5)*  f *Clavien-Dindo grade III or higher*  g *Given insufficient number of degrees of freedom for correction for all possible confounders, only confounders leading to a 10% change in OR were included for analyses. Hospital ID as random effect was added to the model in case the log-likelihood ratio test showed a better fit compared to the original univariable model.*  h *Includes*: *postoperative bleeding (excluding intraluminal), recurrent nerve injury, iatrogenic intestinal injury, gastric tube necrosis, iatrogenic tracheal or bronchial injury, persistent air leakage requiring drainage > 10 days postoperatively, chyle leakage, anastomotic leakage, intraoperative complications.*  i *All postoperative complications not mentioned in H.*  j *Postoperative complication leading to a reintervention, mortality, or prolonged length of hospital stay (>21 days).*  k *Patients with a complicated postoperative course eventually dying in hospital or in first 30-days postoperatively.*  l *Patients undergoing a radical, curative resection with at least 15 resected lymph nodes, without intraoperative complication, severe postoperative complication*^F^*, reintervention, readmission (to the ICU), mortality and a length of hospital stay shorter than 21 days.* | | | | | | | | | | | | |

| Supplementary file Table 5. Impact of weekday of surgery, Monday-Tuesday versus Wednesday-Friday, on short-term surgical outcomes after open or hybrid oncologic esophagogastric surgery in 2015-2019. | | | | | | | | | | | | |
| --- | --- | --- | --- | --- | --- | --- | --- | --- | --- | --- | --- | --- |
|  |  |  | Esophageal carcinoma | | | |  |  | Gastric carcinoma | | | |
|  |  |  |  | | | |  |  |  | | | |
|  | Weekday | *Corrected for* | Outcome/N | OR^a^ | 95% CI^b^ | P-value |  | *Corrected for* | Outcome/N | OR^a^ | 95% CI^b^ | P-value |
| **Complications** (yes) | Mon-Tue (ref)  Wed-Fri | *Random effect of hospital identification number^c^* | 238 / 363  264 / 435 | 1  1.05 | 0.77 – 1.47 | 0.769 |  | *No relevant confounders identified^c^* | 181 / 364  113 / 249 | 1  0.85 | 0.61 – 1.17 | 0.312 |
| **Severe complications**^d^  (yes) | Mon-Tue (ref)  Wed-Fri | *Random effect of hospital identification number^c^* | 106 / 363  122 / 435 | 1  1.07 | 0.76 – 1.51 | 0.693 |  | *No relevant confounders identified^c^* | 76 / 364  54 / 249 | 1  1.05 | 0.71 – 1.55 | 0.810 |
| **Technical complications**^e^ (yes) | Mon-Tue (ref)  Wed-Fri | *Random effect of hospital identification number^c^* | 118 / 363  119 / 435 | 1  0.97 | 0.69 – 1.38 | 0.884 |  | *No relevant confounders identified^c^* | 59 / 364  33 / 249 | 1  0.79 | 0.49 – 1.24 | 0.315 |
| **Medical complications**^f^  (yes) | Mon-Tue (ref)  Wed-Fri | *No relevant confounders identified^c^* | 139 / 363  160 / 435 | 1  0.94 | 0.70 – 1.25 | 0.905 |  | *No relevant confounders identified^c^* | 138 / 364  87 / 249 | 1  0.88 | 0.63 – 1.23 | 0.453 |
| **Anastomotic leakage**  (yes) | Mon-Tue (ref)  Wed-Fri | *Random effect of hospital identification number^c^* | 63 / 363  60 / 435 | 1  1.00 | 0.65 – 1.54 | 0.992 |  | *No relevant confounders identified^c^* | 26 / 364  17 / 249 | 1  0.96 | 0.50 – 1.79 | 0.891 |
| **Complicated postoperative course^g^**  (yes) | Mon-Tue (ref)  Wed-Fri | *Random effect of hospital identification number^c^* | 108 / 363  114 / 435 | 1  0.97 | 0.69 – 1.37 | 0.874 |  | *No relevant confounders identified^c^* | 79 / 364  57 / 249 | 1  1.08 | 0.73 – 1.58 | 0.708 |
| **Failure to rescue^h^**  (yes) | Mon-Tue (ref)  Wed-Fri | *Anastomotic location^c^* | 15 / 108  18 / 114 | 1  1.28 | 0.60 – 2.80 | 0.525 |  | None^i^ | 18 / 79  9 / 57 | 1  0.64 | 0.25 – 1.51 | 0.315 |
| **Surgical radicality**  (micro- and macroscopically radical) | Mon-Tue (ref)  Wed-Fri | *No relevant confounders identified^c^* | 341 / 363  412 / 435 | 1  1.33 | 0.71 – 2.49 | 0.370 |  | *No relevant confounders identified^c^* | 330 / 364  222 / 249 | 1  1.01 | 0.58 – 1.80 | 0.975 |
| **Resected lymph nodes**  (≥15) | Mon-Tue (ref)  Wed-Fri | *Hospital volume, esopha-gectomy type and random effect of hospital identification number^c^* | 300 / 363  345 / 435 | 1  1.24 | 0.79 – 1.93 | 0.352 |  | *Clinical N-stage and random effect of hospital identification number^c^* | 308 / 364  199 / 249 | 1  1.25 | 0.75 – 2.09 | 0.399 |
| **Reintervention**  (yes) | Mon-Tue (ref)  Wed-Fri | *Random effect of hospital identification number^c^* | 88 / 363  95 / 435 | 1  1.01 | 0.70 – 1.45 | 0.963 |  | *No relevant confounders identified^c^* | 64 / 364  51 / 249 | 1  1.21 | 0.80 – 1.82 | 0.367 |
| **Length of ICU stay**  (≥2 days) | Mon-Tue (ref)  Wed-Fri | *All^j^* | 183 / 363  227 / 435 | 1  1.27 | 0.83 – 1.94 | 0.274 |  | *Random effect of hospital identification number^c^* | 81 / 364  58 / 249 | 1  1.03 | 0.67 – 1.58 | 0.890 |
| **30-day/in-hospital mortality**  (yes) | Mon-Tue (ref)  Wed-Fri | *Anastomotic location^c^* | 15 / 363  20 / 435 | 1  1.26 | 0.63 – 2.60 | 0.515 |  | *No relevant confounders identified^c^* | 18 / 346  10 / 249 | 1  0.80 | 0.35 – 1.74 | 0.589 |
| **30-day readmission**  (yes) | Mon-Tue (ref)  Wed-Fri | *No relevant confounders identified^c^* | 46 / 363  54 / 435 | 1  0.99 | 0.65 – 1.52 | 0.974 |  | *No relevant confounders identified^c^* | 48 / 346  31 / 249 | 1  0.96 | 0.59 – 1.55 | 0.872 |
| **Textbook outcome^k^**  (yes) | Mon-Tue (ref)  Wed-Fri | *All^j^* | 151 / 363  193 / 435 | 1  1.11 | 0.80 – 1.56 | 0.532 |  | *No relevant confounders identified^c^* | 181 / 346  116 / 249 | 1  0.88 | 0.64 – 1.22 | 0.445 |
| a *Odds Ratio*  b *95% Confidence interval*  c *Given insufficient number of degrees of freedom for correction for all possible confounders, only confounders leading to a 10% change in OR were included for analyses. Hospital ID as random effect was added to the model in case the log-likelihood ratio test showed a better fit compared to the original univariable model.*  d *Clavien-Dindo grade III or higher*  e *Includes*: *postoperative bleeding (excluding intraluminal), recurrent nerve injury, iatrogenic intestinal injury, gastric tube necrosis, iatrogenic tracheal or bronchial injury, persistent air leakage requiring drainage > 10 days postoperatively, chyle leakage, anastomotic leakage, intraoperative complications.*  f *All postoperative complications not mentioned in E.*  g *Postoperative complication leading to a reintervention, mortality, or prolonged length of hospital stay (>21 days).*  h *Patients with a complicated postoperative course eventually dying in hospital or in first 30-days postoperatively.*  i Asa score, tumor location and hospital volume were confounders *but given the small group sizes and small number of degrees of freedom multivariable regression was not possible. Univariable results are presented.*  j *Corrected for: gender, age, preoperative weight loss, BMI, Charlson Comorbidity Index, ASA-score, previous esophageal or gastric surgery, tumor location, histology, clinical Tumor stage, clinical Node stage, neoadjuvant therapy, salvage surgery, hospital volume, year of surgery, type of esophagectomy, location of anastomosis and hospital identification number as random effect factor.*  k *Patients undergoing a radical, curative resection with at least 15 resected lymph nodes, without intraoperative complication, severe postoperative complication*^d^*, reintervention, readmission (to the ICU), mortality and a length of hospital stay shorter than 21 days.* | | | | | | | | | | | | |
